# Complete Genome Sequence of a Streptococcus pyogenes Serotype M12 Scarlet Fever Outbreak Isolate from China, Compiled Using Oxford Nanopore and Illumina Sequencing

**DOI:** 10.1128/genomeA.00389-18

**Published:** 2018-05-03

**Authors:** Yuanhai You, Yongjun Kou, Longfei Niu, Qiong Jia, Yahui Liu, Mark R. Davies, Mark J. Walker, Jiaqiang Zhu, Jianzhong Zhang

**Affiliations:** a State Key Laboratory of Infectious Disease Prevention and Control, Collaborative Innovation Center for Diagnosis and Treatment of Infectious Diseases, National Institute for Communicable Disease Control and Prevention, Chinese Center for Disease Control and Prevention, Beijing, China; b Beijing XinhuiPro Science and Technology Co. Ltd., Beijing, China; c Department of Microbiology and Immunology, Peter Doherty Institute for Infection and Immunity, The University of Melbourne, Melbourne, Victoria, Australia; d School of Chemistry and Molecular Biosciences and Australian Infectious Diseases Research Centre, The University of Queensland, Brisbane, Queensland, Australia

## Abstract

The incidence of scarlet fever cases remains high in China. Here, we report the complete genome sequence of a Streptococcus pyogenes isolate of serotype M12, which has been confirmed as the predominant serotype in recent outbreaks. Genome sequencing was achieved by a combination of Oxford Nanopore MinION and Illumina methodologies.

## GENOME ANNOUNCEMENT

Scarlet fever has been reported as resurging in several countries since 2011. The epidemic was first reported in China and other Asian countries. Recent studies have correlated the current global outbreak of scarlet fever to the transmission of mobile genetic elements in Streptococcus pyogenes (1–11). Global surveillance of group A streptococci containing these elements is necessary, but it is difficult to obtain complete sequences of these bacteriophages and integrative and conjugative elements, as they are large and may contain repetitive sequences that are not easily traceable using Illumina short-read technology. Despite the multitude of draft genome sequences available, there is no available completed genome sequence of a scarlet fever isolate from mainland China.

Here, we present the complete genome sequence of Streptococcus pyogenes serotype M12 isolate TJ11-001 using Oxford Nanopore Technologies (ONT) and Illumina sequencing platforms. This strain was isolated in 2011 from a patient with scarlet fever in northern China, where there has been a high incidence of disease. TJ11-001 was cultured on Trypticase soy agar containing 5% sheep blood at 37°C for 24 h. Genomic DNA was extracted using a Qiagen minikit and then quantified and quality controlled using Qubit 2.0 and Nanodrop software, respectively. The ONT library was prepared using the manufacturer’s ligation sequencing kit (SQK-LSK108). Sequencing was undertaken using a MinION device integrated with a flow cell (FLO-MIN 106, R9.4). A total of 1.5 μg of genomic DNA was used to perform library construction according to the ONT protocal. Finally, the prepared library was dropped into the flow cell for MinION sequencing. MinKNOW software was used to perform a quality check on the flow cell before running the sample. TJ11-001 was also sequenced using an Illumina HiSeq 2500 instrument. Paired-end libraries with 433-bp mean insertions were generated, and the read lengths were 125 bp. Unicycler (12) was used for complete genome sequence assembly, based on ONT long reads and Illumina short reads.

Illumina sequencing generated 2,679,224 reads and 335 Mb of high-quality raw data. *De novo* assembly generated 25 contigs of 1,904,680 bp length. The MinION sequencing generated 18,610 reads and 286 Mb of raw data during the first 3 h and was then stopped. *De novo* assembly in combination with the Illumina short reads and ONT long reads generated a single contig with a length of 1,939,733 bp and a G+C content of 38.5%. In total, 1,988 coding sequences were predicted. Genome sequence alignment shows that TJ11-001 harbors four mobile genetic elements, including ϕHKU.ssa and ICE-HKU937, which may both play an important role in the clonal expansion of M12 group A Streptococcus (GAS) species, which cause scarlet fever.

This TJ11-001 sequence is the first complete genome sequence from a scarlet fever patient linked to the epidemic in China, and it will serve as a resource for molecular epidemiological investigations of this outbreak. Our study suggests that the portable Nanopore sequencer can be employed for the surveillance of mobile genetic elements within the S. pyogenes population. Such surveillance will be beneficial for the investigation of the current global scarlet fever epidemic.

### Accession number(s).

The TJ11-001 sequence assembly was deposited in DDBJ/EMBL/GenBank under the accession number CP028148, with MinION and Illumina raw data deposited under BioProject number PRJNA448030 and BioSample number SAMN08818899.
